# Body Schema plasticity of the arm: a systematic review of the methods and tasks

**DOI:** 10.3389/fpsyg.2025.1458409

**Published:** 2025-07-01

**Authors:** Anna Zigrino, Pierpaolo Zivi, Fabio Ferlazzo, Stefano Sdoia

**Affiliations:** Department of Psychology, Sapienza University of Rome, Rome, Italy

**Keywords:** Body Schema, plasticity, peripersonal space, agency, proprioception

## Abstract

**Introduction:**

The Body Schema represents the body in a way that allows for dynamic adaptation and integration of motor functions. It receives signals from different sensory modalities, including proprioception, vision and touch, to continuously update to plan and execute body movements. Moreover, it works synergistically with the Peripersonal Space, to enable efficient interactions with the outside world. To do so, the Body Schema temporarily alters itself, plastically adapting to different environmental requests. This work aims at reviewing and categorizing the most commonly used methods in the study of Body Schema, as an attempt to better understand its plasticity and adaptability in different circumstances.

**Methods:**

Two prominent databases, namely Scopus and PsychInfo, were consulted. The eligibility criteria included studies conducted on humans, wherein the population was not clinical. Finally, studies were included in which the Body Schema was considered in isolation, without comparison to other body representations.

**Results:**

The selected papers were grouped into ten different categories, illustrating the various ways in which the Body Schema has been investigated.

**Discussion:**

Different methods to study the plasticity of the Body Schema are discussed. Moreover, it is hypothesized that two common denominators are fundamental for granting the Body Schema its functions: proprioception and sense of agency. Clinical and future research implications are discussed.

## 1 Introduction

Effective navigation of the environment depends on precise motor skills and spatial awareness, that is understanding one's position relative to objects and landmarks. Think about when you have to take the route to the bus stop, or grab your favorite mug from the cupboard, or again, when you see a friend of yours and you want to wave hello. To do all these things, humans need to have a functional representation of the body in space and of their surroundings so that fine motor planning and execution can take place successfully (Newcombe, 2019). Said functional representation of the body is the Body Schema (BS). The BS is a concept of interdisciplinary interest, that has been approached by various disciplines, including philosophy (Merleau-Ponty, 1945), psychology (Reed and Farah, 1995), neuroscience (Bertuccelli et al., 2024), sports medicine (Rodríguez-Camacho and Alvis-Gomez, 2017), and robotics (Hoffmann et al., 2010). In cognitive psychology and neuroscience, the BS is described as a guidance system for actions and body control. Philosopher Merleau-Ponty made a great contribution to the ontological relevance of the BS in the realm of phenomenology. In his ambitious work, the Phenomenology of Perception, about clearing ambiguities regarding phenomenology, philosopher Merleau-Ponty examines the mind-body problem by rejecting the dualistic, mutually exclusive Cartesian approach of res cogitans and res extensa. Instead, he embarks on Husserl's journey of demonstrating how mind and body are both active parts of the subjective experience. Indeed, if the body is no longer regarded as an object or a machine that merely responds to commands, but rather a “vehicle,” an “agent,” or a “fulcrum,” as the author asserts, then the core of an individual's experience in the world depends on the reliability attributed to the body and on the possibility to transform it (Halák, 2018).

In the cognitive psychology and neuroscience language, this concept translates into the BS being a guidance system for actions and body control. More specifically, it serves two main purposes:

- During the initial stages of life, the BS provides fundamental motor functions that guarantee the infant's survival (see hand-mouth coordination, Gallagher et al., 1998).
- During the lifespan, it continuously updates for optimal motor planning and execution of movements (Di Vita et al., 2016).

The one thing that these different domains seem to agree on is the definition of the BS as a more dynamic representation of body posture (Head and Holmes, 1911).

Though it is worth mentioning that at some point the BS has even been argued against its own existence (Cardinali et al., 2009b), today there is a large consensus among researchers about the BS being an important component of body representation. It stems from afferent and efferent inputs from the skin and proprioceptive receptors, whose signals are sent to the primary somatosensory cortex. In other words, it indicates an ongoing, primarily unconscious integration of successive proprioceptive signals (Maravita and Iriki, 2004). These distributions of signals resemble the infamous Penfield's “homunculus,” with some areas of the body having more receptors and therefore being more largely represented. Such primary information is then processed to construct a more cognitive and complex representation of the body, from which the BS emerges. It facilitates action planning and execution by estimating the body's current position and predicting its desired position upon movement completion (Haggard and Wolpert, 2005). Apart from proprioception, it has been demonstrated that other sensory modalities contribute to its construction. Indeed, vision and touch appear to be significant elements of the overall process (Farnè et al., 2000; Pavani et al., 2000).

The BS is both stable, maintaining consistent body part relations to ensure a continuous sense of self, and plastic, constantly reshaping its boundaries in anticipation of movement, momentarily expanding or contracting to integrate the necessary motor adjustments for seamless action. This plasticity attribute of the BS proves to be essential for interacting with the environment and adjusting to changes such as tool use, injury or motor tasks. Indeed, the BS can respectively expand to incorporate objects as part of the body, reorganize following injuries and lead to phenomena like phantom limb sensations or, conversely, prosthetics integration, and finally, it can alter itself to adapt to immediate change, like planning and executing the trajectory of a reaching or grasping movement.

Yet, some aspects of BS plasticity remain unclear. For starters, it is very common that researchers use different operational definitions and experimental paradigms, so that it becomes challenging to compare results across studies. Moreover, there is not a sufficient amount of studies that focus on the difference between short-term, transient body adaptations and long-lasting, structural ones. The literature would certainly benefit from more longitudinal studies to clear existing ambiguities. Lastly, we should be careful to generalize the findings in the literature since they often lack ecological validity and show mixed results when it comes to clinical populations.

With this review we aim at giving an overview of the methods and tasks commonly used in the literature to study the plasticity of the BS, in an attempt to rethink the current procedures and possibly develop a novel, more integrated and precise approach.

It is worth mentioning that the literature distinguishes other types of body representation, like the Body Image (BI) and the Body Model (BM), each serving its specific purpose. We will briefly describe them since it is important to firstly disambiguate them, secondly because in the past, the concepts of BS and BI have been confused (Gallagher, 1986).

The BI is a perceptual, conscious component, defined as a complex psychological construct involving the subjective perception of body appearance and its associated feelings, beliefs and thoughts (de Vignemont, 2010). These beliefs can be either good or bad, and everything in between, and can change with time. The BM is a stored model of the body's metric properties, such as size and shape. It serves the purpose of being referred to by the BS, through the combination of perceptual abilities, like tactile size perception, with position sense (Longo and Haggard, 2012; Longo, 2015a). It is a different construct than the BS, since it has been shown that the BM is prone to be systematically distorted. This means that different body parts seem to be perceived as larger and shorter than their effective proportions. These findings emerged from studying the hand, which was shown to be misperceived in both the horizontal and the vertical axis, in a way that resembles the primary somatosensory homunculus of Penfield (Longo and Haggard, 2010; Peviani et al., 2021). Similar results have been replicated on the forearm (Longo, 2017), leg (Stone et al., 2018), and face (Mora et al., 2018). The systematic distortion of hand proportions is quite controversial since it raises the question of how the human body can execute precise and skilled movement while anchored to such a distorted representation (for a discussion see Bassolino and Becchio, 2023). A convincing explanation for this question has not been formulated yet. In our opinion, there is a clear overlap between the functions and the characteristics of the BS and the BM. It seems to us that the two body representations work together to ensure effective motor planning and execution in space. Since the distortions of the BS in a healthy body are reversible, but the same cannot be said for the distortions of the BM, an important area of study, that is the main interest of this review, concerns BS plasticity and the methods used to study it.

Since we are concerned with how the BS plastically adapts to motor planning and execution, the present work will not focus on the concept of body image nor body model. Instead, this work aims at reviewing and categorizing the most commonly used methods in the study of BS, as an attempt to shed some light on the circumstances and modalities in which the plastic properties of the BS emerge.

From a methodological standpoint, it has been demonstrated that bodily illusions, such as the Rubber Hand Illusion (RHI), the Mirror Box Illusion (MBI), and tool-use paradigms, can alter the BS by extending its boundaries to incorporate external objects like tools (Maravita and Iriki, 2004). In clinical research, these effects are observed both in healthy individuals (Rossetti et al., 2020) and in patients with neurological conditions such as hemiplegia (Tosi et al., 2018). While these alterations are typically temporary, they can become more enduring—either beneficially, as seen in motor skill development or rehabilitation after injury, or negatively, as in cases where a limb is permanently lost. Such changes are gradually reinforced when tasks are performed (or neglected) frequently, allowing the updated BS to be retained for future use without requiring constant recalibration.

These findings, in conjunction with the philosophical conceptualizations discussed above, place the concept of BS within the vast framework of the Embodied Cognition, which posits that cognitive processes are inextricably rooted in the body's interactions with the world, with the body itself serving as a pivotal conduit for shaping the mind (Borghi et al., 2013). This makes the study of BS of great importance for the scientific community. Understanding how the body interacts with space and objects and how it continuously updates its position and coordinates to better plan for action means gaining a better comprehension of how perception works and, therefore, how sometimes perception fails to properly work. In this specific case, think about how relevant it would be to shed light on the neural bases of BS disorders observed in neurological patients suffering from neglect, apraxia, autotopagnosia, or phantom limb experiences (Rumiati et al., 2010).

One of BS main properties is the ability to plastically adapt to motor planning, registering and updating body position in space. This ability proves to be essential on an evolutionary level, since it gives the advantage of continuously adapting to one's surroundings, of making precision grips and of properly interacting with others, just to mention a few. Since this type of body representation accounts for motor planning, and shows its marked malleability, it is also a concept that is highly interconnected with Peripersonal Space (PPS) and Sense of Agency (SoA). PPS has been defined as the space surrounding the body, where physical interactions with elements of the environment take place (Bogdanova et al., 2021). In sum, it is the space of action. It depends on the activity of multisensory neurons in the fronto-parietal network, including the premotor cortex and the posterior parietal cortex. Studies on humans have shown that auditory or visual stimuli presented in the PPS modulate the excitability of the hand representation in the motor cortex (Avenanti et al., 2012). These neurons exhibit responsiveness to tactile stimuli applied directly to specific body regions, as well as to visual and/or auditory stimuli presented in close proximity to those same body regions. Hence PPS has a sensory, as well as a motor function (Serino et al., 2009). These two representations, BS and PPS, work synergically to allow efficient interactions with the outside world, through the temporary modification of BS proportions.

The sense of agency (SoA) refers to the subjective experience of controlling your own actions and, through them, producing some effects on the outside world. According to Gallagher (2000), SoA determines the sense of self and is closely related to motor control processes, of which one is the BS. Furthermore, the SoA can be disrupted in several psychiatric and neurological conditions, like schizophrenia (Moore and Fletcher, 2012) and depersonalization (Ciaunica et al., 2024).

To our knowledge, literature has not provided an extensive study on BS plasticity yet. Our goal in this review is to give an overview of the topic by pointing out different ways in which BS plasticity has been studied. We will proceed to highlight, through the scoping of two well-known databases, how it is possible to observe BS plasticity in a non-clinical population. After a display of all the data collected, practical and clinical implications are discussed.

## 2 Method

### 2.1 Inclusion and exclusion criteria

In an effort to retrieve the appropriate articles, a search was conducted for studies that discussed the various forms of body representation (BR). This step was needed to retrieve the largest amount of records concerning the BS, since lots of papers consider it together with other constructs, we decided to just keep articles in which the BS was properly assessed, since they were the most informative. We also just considered works conducted on humans. Moreover, we decided to exclude anything regarding eating disorders and body dysmorphia, keeping in mind that this review aims at categorizing what variables influence the plasticity of the BS in a non-clinical population. In fact, “eating disorder” and “disordered” were excluded from the script in an attempt to isolate all the studies that considered the BS in relation with psychiatric diseases. Finally, once the screening was finished, we grouped the remaining papers by means of what their main variable of interest was, so that we got some papers about tool embodiment, some others about expertise and so on.

### 2.2 Sources

Following these guidelines, the search string was elaborated. It was meant to scope two big databases such as Scopus and PsychInfo. The search string we came up with is the following:

(“body schema” OR “body image” OR “body representation” OR “body distortion”) AND (“peripersonal space” OR “motor programming”) AND NOT “eating disorder” AND NOT “disorder”

**Figure 1:**
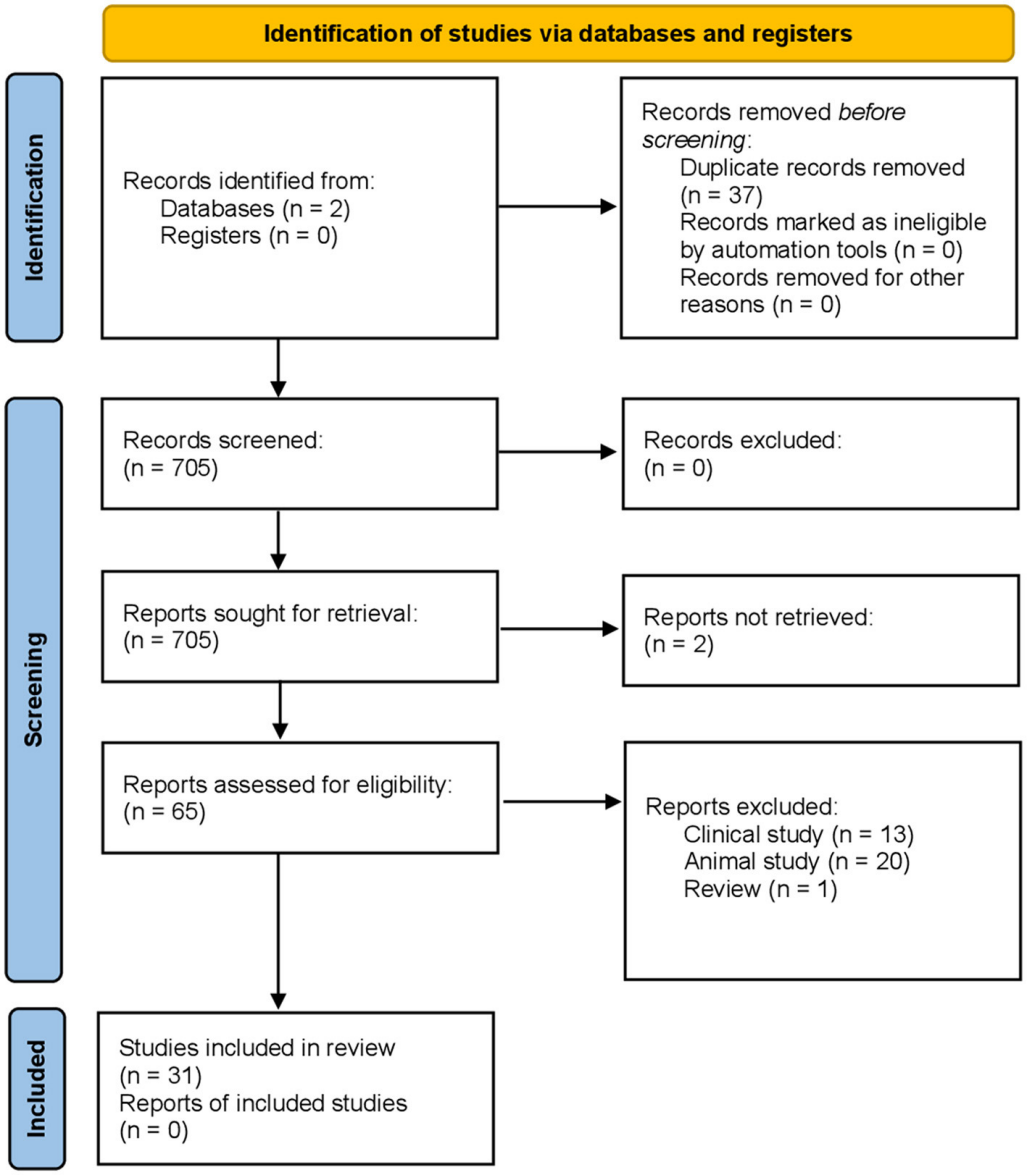
PRISMA 2020 flow diagram for new systematic reviews which included searches of databases and registers only.

The last search conducted on Scopus on the 11^th^ of June 2024 held 644 results, while the last search on PsycInfo was conducted on the 11^th^ of June as well and held 99 results. On both databases, we limited the search to published articles and the field categories of “psychology” and “neuroscience.” A first selection was made by reading titles and abstracts. Then, we proceeded by reading the full text of the remaining articles and deciding whether to keep them or not through the analysis of the methods and procedures used (Figure 1). We chose to consider studies that directly measured the BS, since a lot of records contained comparisons with body image or used methods like the Rubber Hand Illusion which is usually a way of observing body ownership and evaluating perceptive illusions more than the BS. We decided it was best to keep records in which the Rubber Hand Illusion was used to properly discuss the recalibration of the BS (e.g., Lewis and Lloyd, 2010). We excluded all of those studies in which the RHI was implemented for other experimental purposes.

### 2.3 Risk of bias

The JBI checklist (Aromataris et al., 2015) risk of bias tool for analytical cross-sectional studies was adapted to perform a quality assessment of the selected studies. The JBI consists of eight items, which are the following: (Q1) “Were the criteria for inclusion in the sample clearly defined?,” (Q2) “Were the study subjects and the setting described in detail?,” (Q3) “Was the exposure measured in a valid and reliable way?,” (Q4) “Were objective, standard criteria used for measurement of the condition?,” (Q5) “Were confounding factors identified?,” (Q6) “Were strategies to deal with confounding factors stated?,” (Q7) “Were the outcomes measured in a valid and reliable way?,” (Q8) “Was appropriate statistical analysis used?”

Each item was assigned a score of 1 for “Yes” responses and 0 for the remaining “No,” “Unclear,” or “Not applicable.” Then, we summed the scores for each “Yes” response on each item and evaluated the quality of each study as high (≥6); moderate (5–4), or Low ( ≤ 3). On 31 total studies, 10 showed moderate quality, none showed a low quality and 21 showed a high quality assessment.

**Figure 2:**
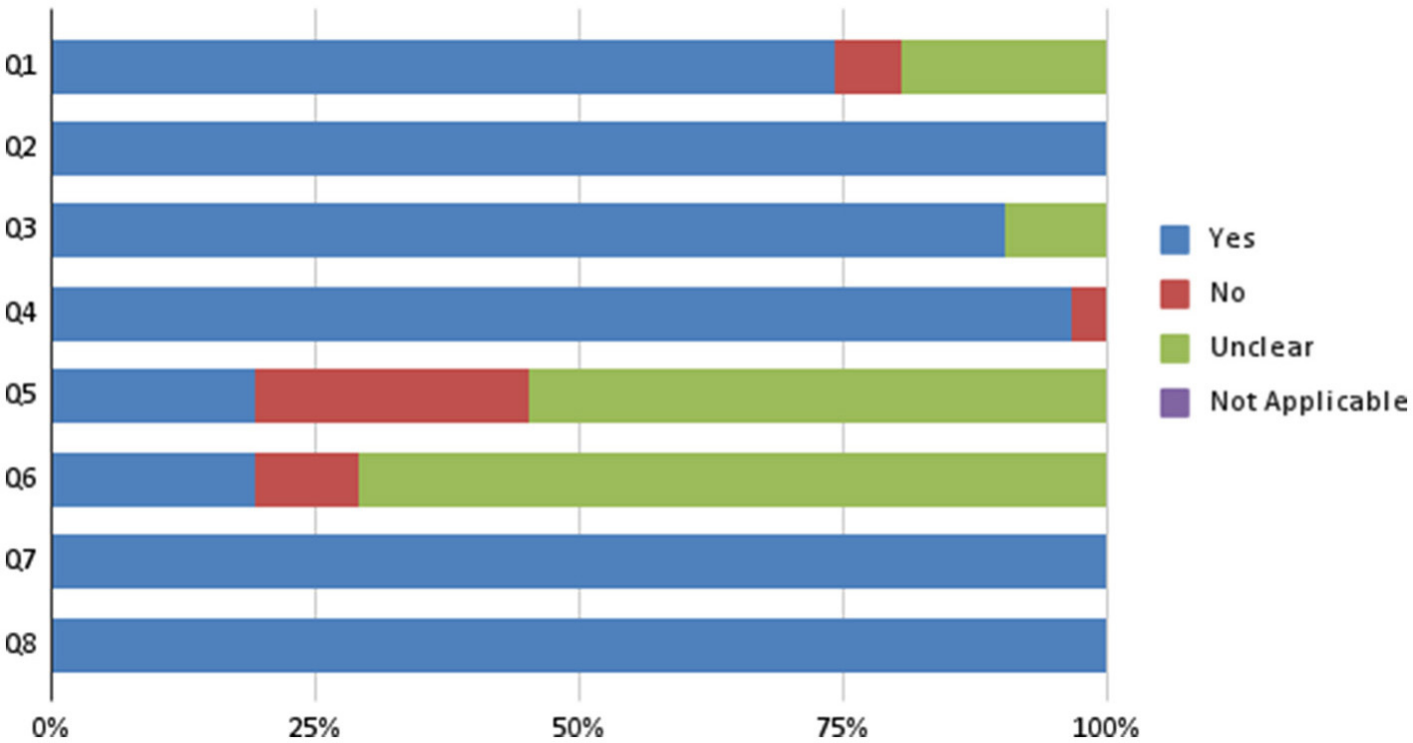
Risk of bias assessment-JBI.

Figure 2 summarizes the quality assessment conducted on the selected studies.

## 3 Results

**Figure 3:**
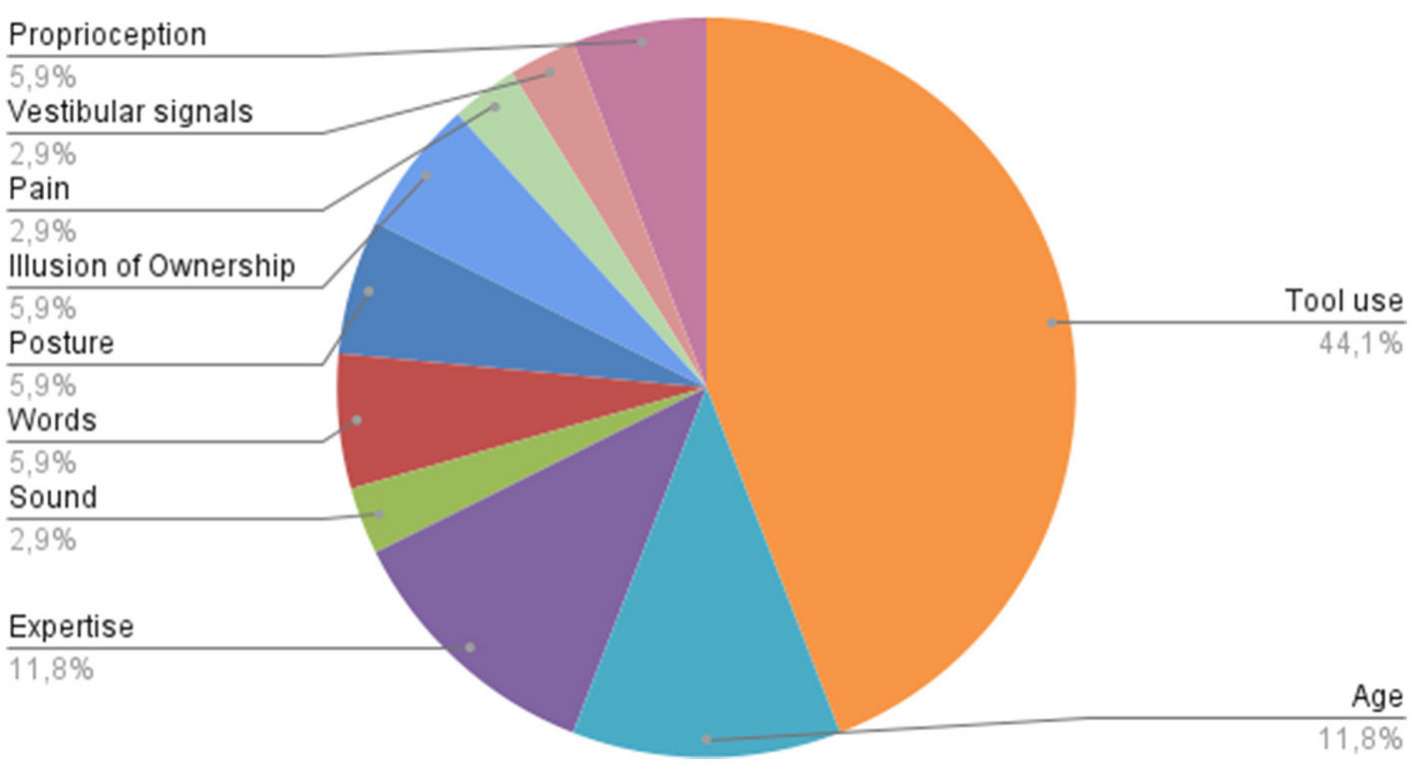
Variables tested to investigate the Body Schema.

After the first screening, 65 records were gathered. We then proceeded to read the full texts to further select what papers met the eligibility criteria. We were left with 31 final articles. We were also able to divide them based on the main topic of research, so that we grouped them into 10 different categories that will be individually discussed and are summarized in Figure 3.

The records screened are all experimental, including two pretest-posttest (Fonseca et al., 2014; Rademaker et al., 2014). They were conducted between 2009 and 2022 and they are very heterogeneous regarding the tasks used and the methods. The Mental Rotation Task was employed by one study (Liu et al., 2022); then one paper utilized the Posner paradigm (McManus and Thomas, 2020), one paper used the Choice RT (Jovanov et al., 2015), one paper the Speeded Detection Task (Kao and Goodale, 2009), one the Avatar Adjustment Task (Sorrentino et al., 2021), one the Lexical Decision Task (Rueschemeyer et al., 2010) and finally one employed the Grooved Pegboard Test (Thurm et al., 2013).

Moreover, three works chose as tasks the Forearm Bisection Task (Bruno et al., 2019; Romano et al., 2019; Sposito et al., 2012); other two researches focused on the use of a Visual or Tactile Analog Task (Thurm et al., 2013; Canzoneri et al., 2013), a Posture or Size Matching Task was utilized by two studies (Cardinali et al., 2012; Sposito et al., 2012). Also, two papers chose the Image Marking Procedure as elected tasks (Fonseca et al., 2014; Thurm et al., 2013).

The Motor Imagery was utilized in three papers (Baccarini et al., 2014; Tomasino et al., 2012; Brusa et al., 2021). The PPS space was measured using a Audio Tactile Interaction Task by three studies (Canzoneri et al., 2013; Sorrentino et al., 2021; Tajadura-Jiménez et al., 2016). Three papers also chose the RHI to study the BS (Gottwald et al., 2021; Lewis and Lloyd, 2010; Kammers et al., 2010).

The most common tasks used were the Tactile Distance Perception Task, employed in five studies (Sun and Tang, 2019; Miller et al., 2014; Canzoneri et al., 2013; Begum Ali et al., 2014; Lopez et al., 2012), the Grasping/Reaching Task, present in six studies (Rybarczyk and Mestre, 2013; Cardinali et al., 2012; Gianelli et al., 2013; Tang et al., 2016; Kammers et al., 2010; Tajadura-Jiménez et al., 2016), and the Body Landmark Localization Task, chosen in seven studies (Canzoneri et al., 2013; Cardinali et al., 2012; Dupin et al., 2022; Sorrentino et al., 2021; Mora et al., 2021; Longo, 2015b; Lopez et al., 2012).

Finally, eight studies employed a Tool Training phase within their procedures (Sun and Tang, 2019; Romano et al., 2019; Jovanov et al., 2015; Miller et al., 2014; Canzoneri et al., 2013; Sposito et al., 2012; Cardinali et al., 2012; Rademaker et al., 2014).

### 3.1 Tool use

Tool use seems to be the most studied scenario. Fifteen papers out of 34 (44.1%) used a paradigm which involved the use (real, imaginative, or teleoperated) of a tool. Tool use is an extremely important skill developed for survival purposes. It made humans able to reach further, inaccessible places and to protect themselves from threats. Many well-known studies demonstrated that, after tool use, participants tended to consider tools as part of their bodies, perceived their bodies as extended and their BS changed (Iriki et al., 1996; Cardinali et al., 2009a, 2011). This phenomenon is also known as tool embodiment. Other studies went way beyond, demonstrating an actual incorporation of the tool used into the BS. In one study, the authors used an arm bisection paradigm and asked the participants to identify the midpoint of their arms before and after a training phase with a tool. Results showed that participants perceived the midpoint of their arm to be more distal after the training phase, demonstrating that the incorporation of the tool made them think that their arm was longer than it actually was (Sposito et al., 2012).

An interesting discussion revolves around what type of tools are able to be incorporated into the BS. An article from Liu et al. (2022), posits that smartphones, as special kinds of objects that we manipulate every day, have the ability to be integrated into our body representation even if they do not literally extend the boundaries of our own bodies. Through a mental rotation task, in which participants had to decide whether the stimulus (a smartphone, non-smartphone, and a hand doing the gesture of holding a phone), was held by a right or a left hand. Participants showed better cognitive processing and motor planning when presented with smartphone in hand stimuli. The explanation given by the authors is that they may have benefited from their modified BS, which had been modified to include the smartphone, consequently making their reaction times quicker.

Speaking about objects, it was also demonstrated that a rake can be successfully incorporated into the BS. In this study, participants had to perform a choice reaction time task before and after training different limbs (hand and foot) with a rake object. Results show support toward the limb-specific hypothesis, since the rake was correctly integrated into the body schema of the hand but not the foot, showing the importance of body-part compatibility for a successful integration of objects (Jovanov et al., 2015).

McManus and Thomas (2020), investigated if the active use of a controlled tool is sufficient to introduce a mental representation of a new zone of PPS around the tool's functional area. What they found through a paradigm using a handheld and a remotely operated tool was that participants showed the advantage of incorporating into the BS objects that were hand-held, while the same could be said about remotely operated ones. Their proposed explanation for these results is that active use of a tool alone is not sufficient for tool embodiment, and that there might be other mechanisms that grant the ability to incorporate objects into the BS. The same question has been approached from a slightly different perspective by Bruno et al. (2019). Their study's main aim was to investigate how tool action may shape body representation by contrasting two views (mere motor execution of tool action vs. the coexistence between goal representation and bodily movements). In doing this, they needed a pair of situations that differ in that one involved the representation of the tool action goals and motor programs, whereas the other did not. To do so they implemented a set-up in which participants performed the task with their forearm attached to a robotic limb. They had to either move the robotic limb through their own movement (robot off) or stay relaxed and let the robotic movements guide their arm (robot on). The authors found no modulation in perceived arm length (through a forearm bisection task) when the robot was passively moving participants' arms, which, on the contrary, they found in the other condition. Their findings indicate that motor processes and representations, involved in planning and monitoring tool action, may also play a crucial role in shaping one's own body metric representation. A different contribution to the study of teleoperated devices predicted that the remote robot would be integrated into the operator's BS only if the topological architecture of the camera-arm robotic system was consistent with human sensorimotor contingencies. To investigate this hypothesis, researchers configured a teleoperated system based on an anthropomorphic architecture resembling human-like characteristics. They compared this configuration with two others that progressively deviated from this natural organization. Their analysis began with what they termed the “anthropometric” condition. Results indicated that participants quickly developed a perception of the capabilities of the remotely controlled arm following minimal training, suggesting rapid remapping of functional space. This finding supports the notion that extensive manipulation of tools is not necessary for such remapping. The study proposes that a teleoperated device can swiftly become integrated into the operator's BS. Conversely, the researchers could not replicate these findings in the “bias” and “side” conditions, indicating that operators struggled to form an accurate representation of the robotic arm's properties (Rybarczyk and Mestre, 2013).

In another study, researchers conducted two experiments to investigate whether tool embodiment could spread to the limb that was not using the tool and whether proprioception is general to all limbs or specific to one limb. The remarkable result they found was that proprioceptive information of one limb can actually be exploited by the other one to expand the BS even though that first limb was not using the tool (Sun and Tang, 2019). One relevant question answered by Romano et al. (2019) as well was whether different tool training could induce specific effects on body metric representation. Their work aimed at investigating the role of user-specific factors in affecting the BS. Using the classic arm bisection task, they tested whether training sessions involving predominantly different motor patterns (i.e., more proximal or distal motor control) could differently modulate body metrics. What they suggested through their results was that the effects of tool embodiment might be influenced by specific actions involved in the training phases. Not only this, they also went further indicating that a crucial role is played by the morphological features of the tool, as well as by the specific way it is used (similar results were replicated by Miller et al., 2014). Moreover, an interesting question regards the possible implications of tool embodiment on perception. One study examined this topic to find out whether or not training people to use a fake hand or a tool (a small five-prolonged garden rake) to manipulate objects would lead to improved detection of visual stimuli presented on the fake hand or the tool after training. The main question that the authors tried to answer is the following: how does the incorporation of inanimate objects into the BS affect the detection of visual stimuli presented on or near those objects? Their findings demonstrate that it is possible to enhance visual detection for stimuli presented on tools and other inanimate objects with training, suggesting that these objects have been incorporated into the BS (Kao and Goodale, 2009). Two studies concentrate on the idea that tool use could induce BS plasticity even in absence of an actual tool, through the use of mental imagery. Both studies provided affirmative outcomes. In particular, one hypothesized that the use of an “appropriate” tool for the respective spatial domain (defined by preference; here: pliers in far space and joystick in near space) would specifically alter neural activity in the Extrastriate Body Area (EBA) indicating an adaptation of the neural representation of the human body by motor simulation of tool use. They were able to successfully show that brain regions involved in body (part) processing are modulated already by motor imagery of tool-use, even in the absence of any overt movement or visual feedback from the acting limb/simulated movement (Tomasino et al., 2012; Baccarini et al., 2014).

Finally, the relationship between BS and PPS was investigated, with the goal of understanding if the use of a tool to manipulate distant objects could not only affect the BS, but also the PPS. The results show a modulation of both the constructs under study, with a sound presented in a far location, in which participants had the chance to practice with a tool, being perceived as closer to the body than it actually was. The authors suggest that these results could be due to an overlap between BS and PPS (Canzoneri et al., 2013).

In summary, it is suggested that some brain regions involved in body part processing are modulated by imagined tool use (two records). Once the tool is being manipulated it is efficiently incorporated into the BS. It seems that different kinds of objects can be incorporated: from rake shaped, grabbing tools to smartphones. There is not a strong consensus among remotely operated objects. One article found a strong integration, two others found less clear results. The effect of tool embodiment seems to be mediated by the specific ways it is used (namely the nature of the task and body part compatibility with the object). Finally, it was shown how training with a tool can induce plastic changes either to the body part holding the tool, and to the space surrounding it, suggesting the presence of a relation between BS and PPS (Table 1).

**Table 1:** Summary of the “tool use” records and their findings.

**Title**	**Authors**	**Year**	**Design**	**Dependent variables**	**Task(s)**	**Key findings**
*Smartphone use can modify the body schema: An ERP study based on hand mental rotation task*	Liu Q., Wu J., Zhou Z., Wang W.	2022	Exp	RT Accuracy ERP	Mental rotation task	Smartphone are a peculiar kind of object that has the ability of being incorporated into the body schema
*Vision is biased near handheld, but not remotely operated, tools*	McManus R.R., Thomas L.E.	2020	Exp	RT Accuracy	Posner	Humans have the advantage of incorporating into the body schema objects that are hand held. The same cannot be said about remotely operated ones
*How Tool-Use Shapes Body Metric Representation: Evidence From Motor Training With and Without Robotic Assistance*	Bruno, V., Carpinella, I., Rabuffetti, M., De Giuli, L., Sinigaglia, C., Garbarini, F., and Ferrarin, M.	2019	Exp	Estimated forearm midpoint (before and after different tool training sessions)	Forearm bisection task Tool-use training with arm attached to a robotic limb (in an “active” or “passive” condition)	Participants significantly overestimated their arm length after the active training session, but not after the passive one. When the tool-use was performed passively (participants remaining relaxed while a robot moved their arm) there was no change in perceived arm length. This might indicate that simply executing a tool action is insufficient to alter body representation; instead, the motor programs must be voluntarily engaged and internally represented
*Tool-Use Training Induces Changes of the Body Schema in the Limb Without Using Tool*	Sun Y., Tang R.	2019	Exp	Estimated distance	Tactile distance perception task Tool-use training (searching with a cane or walking with a cane, both blindfolded)	The proprioceptive information of one limb could be exploited by another limb to extend the body schema
*Different tool training induces specific effects on body metric representation*	Romano D., Uberti E., Caggiano P., Cocchini G., Maravita A.	2019	Exp	Estimated forearm midpoint (before and after different tool training sessions)	Forearm bisection task Distal tool training (hit) Proximal tool training (grab)	The effect of tool embodiment is influenced by specific actions: After the distal tool training, the estimated forearm midpoint shifted toward the shoulder. After the proximal tool training the estimated forearm midpoint shifted more toward the wrist.
*The limb-specific embodiment of a tool following experience*	Jovanov K., Clifton P., Mazalek A., Nitsche M., Welsh T.N.	2015	Exp	RT Accuracy	Choice RT task Tool training task (rake)	Data on this study is consistent with the hypothesis that the tool (rake) was incorporated into the limb-specific region of the body schema through use
*Tool morphology constrains the effects of tool use on body representations*	Miller L.E., Longo M.R., Saygin A.P.	2014	Exp	Perceived distance between two points on a body part (the judgment is about what body part touched had the points furthest apart)	Tactile distance judgment task Tool training task (with a hand-shaped tool or a grabber)	Both function and morphology influence tool-induced representation plasticity
*Tool use imagery triggers tool incorporation in the body schema*	Baccarini M., Martel M., Cardinali L., Sillan O., Farnè A., Roy A.C.	2014	Exp	Latencies and amplitudes of acceleration Velocity and deceleration peaks for the transport component Latency and maximum grip aperture for the grip component	Pre-imagery free-hand grasping task Motor Imagery task Post-imagery free-hand grasping task	Imagery of tool-use may be sufficient to update the representation of the arm length used to execute free-hand movement
*Effect of visuo-manual configuration on a telerobot integration into the body schema*	Rybarczyk Y., Mestre D.	2013	Exp	Pi ratio (ratio of an environmental dimension to a body dimension)	Grasping task with a teleoperated robot	A teleoperated device can rapidly be appropriated and incorporated into the operator's body schema only if seen in an anthropocentric view
*Tool-use reshapes the boundaries of body and peripersonal space representations*	Canzoneri E., Ubaldi S., Rastelli V., Finisguerra A., Bassolino M., Serino A.	2013	Exp	RT Perceived position of sound in space P-forearm (mean probability of reporting the distance on the forearm as longer for all the combinations of inter-point distances) P-second (probability of reporting a longer distance between the two dots in the second stimulus, or in the second stimulus of the target forearm) E–W distance (perceived arm length as the distance between the perceived position of the elbow and the wrist)	Exp 1A: Audio tactile interaction task (PPS) Tactile distance perception task (BR) Tool use training Exp 1B: Visual analog task Tactile analog task Exp 2: Tactile distance perception task Body-landmarks localization task Exp 3: Audio tactile interaction task (PPS) Tactile distance perception task (BR)	A brief training with a tool induces plastic changes both to the perceived dimensions of the body part acting upon the tool and to the space around it, suggesting a strong overlap between peripersonal space and body representation
*Imagined tool-use in near and far space modulates the extra-striate body area*	Tomasino B., Weiss P.H., Fink G.R.	2012	Exp	RT Accuracy Activation clusters through fMRI	Motor imagery task (shortest path clockwise or counterclockwise?)	Brain regions involved in body (part) processing are modulated already by motor imagery of tool-use
*Extension of perceived arm length following tool-use: Clues to plasticity of body metrics*	Sposito A., Bolognini N., Vallar G., Maravita A.	2012	Exp	Estimated forearm midpoint (before and after different tool training sessions) Proprioceptive mismatch (distance of the tips of the middle finger of each arm from the edge of the table, measured blindfolded, pre-training and post-training blocks of line bisection)	Exp 1: Forearm bisection task Tool training Exp 2: Forearm bisection task Tool training (different length tool) Exp 3: Forearm bisection task Tool training Posture matching task	Body space interactions requiring the use of tools that extend the natural range of action, entail measurable dynamic changes in the representation of body metrics
*Grab an object with a tool and change your body: Tool-use-dependent changes of body representation for action*	Cardinali L., Jacobs S., Brozzoli C., Frassinetti F., Roy A.C., Farnè A.	2012	Exp	Velocity peak latencies Deceleration peak latencies	Grasping task (with a mechanical grabber) Size-matching task (with the same tool)	These findings support the hypothesis that the nature of the task, namely whether the tool is used for performing an action or a perceptual judgement, may differentially impact the plasticity of the body schema
*When action is not enough: Tool-use reveals tactile-dependent access to Body Schema*	Cardinali L., Brozzoli C., Urquizar C., Salemme R., Roy A.C., Farnè A.	2011	Exp	Mean length estimation (for the hand and forearm)	Body-landmarks localization task Tool-use training	Motor output is not sufficient *per se*, but has to be coupled with tactually mediated information to guarantee access to the BS
*Enhanced detection of visual targets on the hand and familiar tools*	Kao K.-L.C., Goodale M.A.	2009	Exp	RT Training effect (RT difference score)	Speeded-detection task (with real hand, fake hand, or gardening tool)	An enhanced visual detection can be induced for stimuli presented on tools and other inanimate objects with training, suggesting that these objects have been incorporated into the body schema

### 3.2 Age

How and if the BS changes or adapts during the aging process has been a topic of interest in studies about body representation. One debate that is still open is whether Body representations and PPS are affected by healthy aging. Considering the decline in somatosensory processing that occurs with age, it can be hypothesized that older adults demonstrate greater distortions than younger adults in a body localization task. This phenomenon may be attributed to a potential reduction in the role of afferent bodily signals in updating the stored, distorted model of body parts' metrics. One study revealed more pronounced distortions in body representation in the older adult group compared to the younger group. The results indicated comparable multisensory facilitation for stimuli presented in proximity to the body in both younger and older adults, suggesting a similar PPS representation in the vicinity of the hand. However, older participants exhibited reduced multisensory interaction for stimuli presented at a distance, accompanied by diminished accuracy in auditory localization and slower tactile processing (Sorrentino et al., 2021).

Another study tried to approach the matter from a different angle. There is no direct research on how knowledge of body shape or layout constrains body representation in children. This study was the first to address the role of top-down information, namely internal short- and long-term models of body posture in childhood. There is evidence to suggest that children may possess a greater degree of bodily flexibility than adults. During the developmental period, children's physical and functional capabilities undergo rapid and dynamic changes. In this study, the researchers sought to empirically investigate whether and how posture influences body representation in childhood. To this end, they employed the Rubber Hand Illusion, which enabled them to assess how children's perception of their own bodies was influenced by the match between the viewed and felt limb posture. In the results section they discuss how both the proprioceptive drift measure and subjective ratings demonstrate that bottom-up multisensory information from vision, touch, and proprioception drives body representation for both children and adults. Their main finding is that both adults and children use top-down knowledge of potential body postures to inform their body representation. It has been demonstrated that hand orientation is a significant aspect of the visual model of the body in childhood (Gottwald et al., 2021). A different situation in which BS could show its flexibility is in relation to age and posture. A study investigated the development in early childhood of an ability to represent tactile stimuli with respect to an external spatial frame of reference, and to relocate such tactile locations in external space across changes in the posture of the body. The authors suggest that in early childhood, difficulties in integrating visual cues into the BS can sometimes lead to interference with touch localization when hands and arms are visible. They introduced a Tactile Localization task and found that at age 4, seeing the hands negatively affected tactile localization when the hands were in their typical (uncrossed) positions, meaning when the body and limbs were in a standard arrangement. They interpreted this paradoxical finding as being due to a difficulty in early childhood with combining visual information about the body into the BS, which is subsequently resolved by 5- and 6-year-olds (Begum Ali et al., 2014). The last one focused on the changes to the BS during the lifespan. The aim was to identify quantitative aspects of different factors that could affect spatial hand representation potentially related to sensory and motor functions. Since age is known to affect motor and sensory functions, they hypothesized that age would affect the spatial representation of the hand. The authors further investigated the effect of daily activities and of light touch sensitivity on the metrics of hand representations. They used both a verbal and a tactile localization task, and what they found was that hand representations showed clear deviations from physical hand size: finger length and hand width were smaller than those of the real hand. The size (width) of spatial hand representation decreased as age increased and this was not observed for object representation. Finally, spatial properties of object representation were also partially shared with spatial hand representation characteristics (Dupin et al., 2022).

In summary, 4-year-old children show difficulties performing a tactile localization task on their hand when the hand is visible and in a typical position. This may be due to a still underdeveloped ability to integrate visual sensory information into the BS. The issue seems to be resolved by the 5^th^ year of age. A similar result was replicated using different methods, such as the RHI. Six-year-old children show similar postural constraints to the young adults, indicating that by the 6-year mark, children show similar body representation processes to 20-year-olds. When comparing a group of young adults to older people on a body landmark localization task, substantial distortions in the body representation can be observed in the older adults group. Specifically, hands' width decreased as age increased. Moreover, older adults show less accuracy in identifying auditory stimuli location and slower tactile processing (Table 2).

**Table 2:** Summary of “age” records and their findings.

**Title**	**Authors**	**Year**	**Groups mean age**	**Design**	**Dependent variables**	**Task(s)**	**Key findings**
*Shrinking of spatial hand representation but not of objects across the lifespan*	Dupin L., Cuenca M., Baron J.-C., Maier M.A., Lindberg P.G.	2022	3 groups: 20–39 40–59 60–79	Exp	Averaged hand width and finger length Averaged card width and card length	Body-landmarks localization task (on the hand: physical, tactile, or verbal condition) Pointing at the 4 corners of an imagined credit card (vision or no-vision)	The size (width) of spatial hand representation decreased as age increased and this was not observed for object (card) representation. Spatial properties of object representation were also partially shared with spatial hand representation characteristics
*How aging shapes body and space representations: a comparison study between healthy young and older adults*	Sorrentino G., Franza M., Zuber C., Blanke O., Serino A., Bassolino M.	2021	2 groups: 20–33 53–86	Exp	Estimated width and length (of arm and hand): ratio between real and perceived size Normalized Shape Index (of arm and hand): ratio between estimated width and length Perceived distance (cm): perceived sound when tactile stimulus was given at each of the three temporal delays (D3, D2, and D1) corresponding to three sound distance (D3 $\frac{1}{4}$ far; D2 $\frac{1}{4}$ medium; D1 $\frac{1}{4}$ near)	Body-landmarks localization task Avatar adjustment task Peripersonal space task	Substantial distortions were observed in body representation in the older adults group rather then in the younger. Reduced multisensory interaction for far stimuli, less accurate auditory localization and slower tactile processing were found in older participants
*The Developing Bodily Self: How Posture Constrains Body Representation in Childhood*	Gottwald J.M., Bird L.-A., Keenaghan S., Diamond C., Zampieri E., Tosodduk H., Bremner A.J., Cowie D.	2021	2 groups: 6–7 18–24	Exp	Proprioceptive drift (subtraction of mean baseline pointing position from mean test pointing position) Correct judgments rate d' (hits - false alarms)	Exp 1: RHI Exp 2: RHI (hand rotated 20° anticlockwise) Perceptual judgment task	Both experiments seem to be in line with the authors' “early development scenario”, in which 6- and 7-year-old children show similar postural constraints to young adults. Moreover, orientation of the fake hand appears to be important information that constrains the sense of hand position but not sense of ownership.
*Effects of posture on tactile localization by 4 years of age are modulated by sight of the hands: Evidence for an early acquired external spatial frame of reference for touch*	Begum Ali J., Cowie D., Bremner A.J.	2014	1 group: 4–6	Exp	Accuracy	Tactile localization task (uncrossed-hands, visible; uncrossed-hands, covered; crossed-hands, visible; crossed-hands, covered)	At 4 years, seeing the hands actually had an adverse effect on tactile localization when the hands were in typical (uncrossed) positions. This finding might be due to a difficulty in early childhood in combining visual information about the body into the body schema, which is subsequently resolved by 5- and 6-year-olds

### 3.3 Expertise

A growing body of evidence shows that long-term training and expertise can modulate body representation. For example, magicians appear to have a better perception of finger length (Cocchini et al., 2018), while dancers show an enhanced ability for proprioceptive localization of their hands (Jola et al., 2011). Two of the records focus on Sign Language (SL) speakers as experts in using their hands and face to communicate. One study hypothesized that if the metric representation of hands is associated with the manual workspace and type of expertise, the impact of expertise will be strongly related to the spatial domain. Therefore, SL interpreters will show different metric representations of hands only in near-reaching space, whereas no differences should be found in far-reaching. The authors divided the study into two experiments. In the first one, SL experts considerably outperformed controls at estimating the width of their hands in near-reaching space, but not in far. These results confirmed the link to functional workspace and size representation. Hence, expertise does not modify the mental representation of the hands disregarding localization; instead, it is intrinsically linked to space. Interestingly, this advantage in the representation was specific to the width, not to the lengths. The second experiment yielded comparable outcomes, with SL users demonstrating enhanced accuracy in perceiving the width of face features relative to controls. However, no differences were observed in length perception (Mora et al., 2021). A different study from Brusa et al. (2021) found more controversial data. Their initial question was similar to the previous research: they hypothesized that body representation (BR) in its various components (both motor- and sensory-related)—a significant non-linguistic cognitive index—might explain the varying proficiency levels among individuals learning and using British Sign Language (BSL). Like dancers and magicians, skilled BSL users might have a better body representation, enabling them to use it more effectively for communication. Interestingly, no differences in representing the physical body were found when people used it to communicate in British SL. A performance difference was observed between tango dancers and controls: tango dancers were more accurate in their judgments, especially in the implicit task requiring mental rotation of hands. Dancers exhibited superior perception at the level of single joints, greater proprioceptive acuity, and a more cohesive body representation overall.

Dancers were the elected subjects for yet another research studying whether dancing could influence the BS. Indeed, the act of moving one's body in unison with a partner in accordance with a musical rhythm, coupled with the fluidity induced by dancing, entails the investigation of a multitude of spatial and kinesthetic possibilities. These include the dancer's relationship with their own body, the bodies of others, objects, and the ballroom space itself. The researchers used a projection point test in which participants stood in front of a white wall with a marker in their hands and one of the researchers behind them. The researcher lightly touched one part of the participant's body and the task was for the participant to mark the corresponding point on the wall. The test was conducted before and after a 3-month period, during which half of the subjects attended lectures on body perception while the other half took dance classes. It was observed that participants who practiced ballroom dancing experienced perceptual benefits (Fonseca et al., 2014).

Finally, one study examined the relationship between expertise and second-order integration. Utilizing a novel approach to probing bodily representations, the study expanded upon the recently developed methodology for investigating tool use. The paradigm posits that a cross-modal effect occurs whereby proprioceptive inputs have a profound impact on the visual representation of the body. The authors were able to present evidence that objects (cotton balls) held by a tool (chopsticks) are rapidly integrated into the body representation as indexed by fading of the cotton balls (or second order-extensions) from a positive afterimage. Skillfulness with chopsticks was predictive of more rapid integration of the second-order object held by the tool. They also discovered that extensive practice over several weeks enhanced the level of integration.

In summary, two studies focused on the expertise of SL speakers, either compared to controls or tango dancers. SL speakers seemed considerably more accurate at the hand localization task when in near-reaching space but not far, maybe because of how signing works. When using a different task, namely a mental rotation task, the same advantage of SL speakers could not be found, in respect to tango dancers. Nonetheless, tango dancers showed better accuracy than controls. Other two studies considered the variable “expertise” in a pretest-posttest design, in which subjects trained either their dancing or chopstick use skills. Both demonstrated the benefit of training specific motoric skills for better body perception and object integration (Table 3).

**Table 3:** Summary of “expertise” records and their findings.

**Title**	**Authors**	**Year**	**Type of expertise**	**Design**	**Dependent variables**	**Task(s)**	**Key findings**
*The signing body: extensive sign language practice shapes the size of hands and face*	Mora L., Sedda A., Esteban T., Cocchini G.	2021	Sign Language speakers vs. controls	Exp	Difference between real and perceived hand size Difference between real and perceived face size	Exp 1: Hand localization task (near and far from the body) Exp 2: Face localization task	SL experts considerably outperformed controls at estimating the width of their hands in near-reaching space, but not in far. The groups did not differ in the face localization task
*Talking with hands: body representation in British Sign Language users*	Brusa F., Kretzschmar L., Magnani F.G., Turner G., Garraffa M., Sedda A.	2021	Sign Language speakers vs. tango dancers vs. controls	Exp	RT Accuracy Average movement duration Average duration to image each movement (right and left side separately)	Body esteem scale Mirror letter discrimination task Mental motor chronometry Mental bar movement task	No differences between SL and tango dancers. Significative difference was found between tango dancers and controls in which tango dancers were more accurate in a mental rotation task concerning body parts
*Ballroom dance and body size perception*	Fonseca C.C., Thurm B.E., Vecchi R.L., Gama E.F.	2014	Ballroom dancers vs. controls	Exp pretest-posttest	Body perception index (ideal between 99.4% and 112.3% accuracy)	Image marking procedure (before and after a 3 month period, in which participants participated in either 4 educational lectures or in weekly dance classes)	It was observed that ballroom dancing brought perceptual benefits to those who attended the dance classes vs. those who took the educational lectures. Specifically, participants who attended the dance classes shifted their body perception index from hyper schematic (greater distortions) to adequate.
*Intensive tool-practice and skillfulness facilitate the extension of body representations in humans*	Rademaker R.L., Wu D.-A., Bloem I.M., Sack A.T.	2014	Chopsticks users vs. controls	Exp pretest-posttest	Average trial duration	Chopstick skill test (pretest) After image paradigm (before and after a 4-week chopstick skillfulness training)	Skillfulness with chopsticks was predictive of more rapid integration of the second-order object held by the tool. They also found that extensive practice over a period of weeks augmented the level of integration

### 3.4 Words

Embodied theories of language processing suggest that internal simulations of real interactions with functional objects are mirrored in lexical-semantic representations. In the domain of tool embodiment, it is evident that diverse tools will utilize and extend BS information in various ways. While a cup and a key may both extend the physical length of the hand (i.e., extend PPS), the action goals of cups and keys are clearly distinct, and make very different demands on action planning. When preparing to use a cup, individuals implicitly perceive the spatial relationship between their hand and mouth, utilizing the BS to guide the transfer of the cup (one effector) to the mouth (another effector). In contrast, when preparing to use a key, the individual visually locates the keyhole and positions the hand holding the key in PPS, thereby formulating an action plan with reduced reliance on the BS compared to the cup example. In one study, it is suggested that, in addition to being associated with different movement parameters, objects typically brought to a location on the body (e.g., cup) are relatively more reliant on BS, since the final goal location of the cup (i.e., the mouth) is represented primarily through posture and body coordinates. In contrast, items conventionally transported to a location distant from the body (e.g., a key) exhibit a comparatively greater reliance on visuospatial representations, as the ultimate target location of the key (i.e., a keyhole) is visually perceived. To substantiate this hypothesis, the authors conducted two studies, one behavioral and the other employing neuroimaging techniques. The behavioral study demonstrated that pre-planning a movement along an axis toward or away from the body facilitates the processing of words containing congruent action semantic features (e.g., preparing a movement toward the body enhances processing of the word “cup”). In an fMRI investigation, it was observed that words referring to objects brought toward the body activate brain regions associated with processing information about human bodies—specifically, the extra-striate body area, middle occipital gyrus, and inferior parietal lobe—more prominently than words denoting objects typically moved away from the body. These findings provide compelling evidence that the activation of the BS occurs implicitly during the processing of lexical information (Rueschemeyer et al., 2010). Another paper examines the general limitations of the EG (Embodied-Grounded) and EM (Extended Mind) perspectives, subsequently addressing their particular limitations in comprehending the role of language. It is then suggested that words can be understood as social tools, and explained why this approach would help to reconcile EG and EM views of cognition and to overcome their limitations. Their reasoning takes place considering that words are first encountered as objects. They are peculiar because they implicitly refer to a social and public dimension and because they are immaterial. Only later do they become internalized (Vygotsky, 1962). Even if they are immaterial, the authors suggest that words are both extended and embodied because their use determines a remapping of the relationships between our body, the objects and the space. In sum, words, similarly to tools, lead to a plastic modification of reaching space, even if they cannot be integrated into the BS as tools do. This was demonstrated in a study where the presence of another person in the experimental setting influenced the kinematics of a reach-to-grasp movement, and this effect depended on whether the person was a friend or a non-friend. Moreover, merely hearing or producing first-person pronouns, like “I,” led to faster movements, possibly indicating that language, especially pronouns, can prime action readiness through simulation of the speaker's potential actions.

In summary, these two studies share a common theoretical framework that sees Words As social Tools (WAT) (Borghi et al., 2019). It refers to an additional function that words have, apart from accessing meaning, which enables words to become useful tools to perform actions and change the state of our surrounding environment. Despite using different methods, the two studies suggest the presence of a correlation between certain words and motor planning. From a behavioral perspective, it can be observed that when two people interact with each other, depending on the position and the pronoun used, making a gripping movement could be faster or slower, suggesting that speaking itself can be considered a form of action. Similarly, from a functional perspective, researchers showed that certain areas of the brain involved with human body parts processing are engaged more when reading a word indicating an object brought toward the body than away from the body (Table 4).

**Table 4:** Summary of “words” records and their findings.

**Title**	**Authors**	**Year**	**Design**	**Dependent variables**	**Task(s)**	**Key findings**
*Acting in perspective: the role of body and language as social tools*	Gianelli C., Scorolli C., Borghi A. M.	2013	Exp	Latency of maximal fingers aperture (lMFA) Latency of velocity peak Reaching time (%)	Grasping task (modulated by the person speaking and the pronoun used, “I” or “you”)	When the “other” was positioned to the right of the agent, the proportion of movement time spent on reaching was lower for the pronoun “I” compared to “You.” When the speaker was the “other,” a smaller proportion of movement time was allocated to the reaching phase when using the pronoun “I” rather than “You.” These findings suggest that speaking itself can be considered a form of action, as it increases the visibility and perceived “threat” of the other. This is further supported by the fact that when the other was on the right side and became the speaker, maximal finger aperture occurred earlier, as if language heightened the person's visibility.
*Body schematics: On the role of the body schema in embodied lexical-semantic representations*	Rueschemeyer S.-A., Pfeiffer C., Bekkering H.	2010	Exp	Action-sentence compatibility effect (mean response time and mean performance rates) Activation clusters through fMRI	Lexical decision task Go/no go lexical decision task (stimuli were words that express actions performed away or toward the body)	The behavioral study showed that a priori planning of a movement along an axis toward and away from the body facilitates processing of words with a congruent action semantic feature. The fMRI study showed that words denoting objects brought toward the body enagage the resources of brain areas involved in the processing of information about human bodies relatively more than words denoting objects typically brought away from the body

### 3.5 Posture

Earlier, the significance of tools in daily life was discussed, alongside a description of how they can become incorporated into one's BS through use. One line of research stemmed from considering if and how grasping objects with a tool engages the same motor plan as when fingers are used. In other words, the matter of interest is whether grasping with a tool or with hands shares the same motor plan, also referred to as motor equivalence. One study found that when either fingers or tools are used to perform a grasping action, participants tended to open the relevant effector wider when visual feedback was not available. The authors suggest that the wider finger aperture probably reflects the need to build a margin of error, so that the fingers can close around the target object without poking around or knocking it away. Another article went deeper into the question investigating if the posture of our hands could modulate implicit body representations mediating position sense. The author found that the internal posture of the hands produces rapid modulation of their implicit maps. The researcher instructed participants to either splay their fingers or press them together while engaging in a modified version of the Localization Task (LT). Their observations revealed that when fingers were splayed, the hand was perceived as larger compared to the “pressed together” condition. This finding stands in stark contrast to prior research, which indicated that postural rotations of the hand relative to the body did not affect position sense, tactile size perception, or tactile localization. Thus, it appears that the configuration of the hand, rather than its position or orientation, is the primary driver of these effects.

In summary, when analyzing the effect of hand posture on body representation, the role of visual feedback is important to adjust for effective motor planning and execution. Indeed, regardless of the effector being participants' fingers or a tool, the peak grip aperture was wider when no visual feedback was provided (Table 5).

**Table 5:** Summary of “posture” records and their findings.

**Title**	**Authors**	**Year**	**Design**	**Dependent variables**	**Task(s)**	**Key findings**
*Unusual hand postures but not familiar tools show motor equivalence with precision grasping*	Tang R., Whitwell R.L., Goodale M.A.	2016	Exp	PGA - Peak grip aperture (mm) dPGA (difference in peak grip aperture between closed-loop and open-loop trials)	Grasping task –> 5 different grasps, either a finger combination or using objects (open-loop and closed-loop, objects of different sizes)	No matter if fingers or tools are used to grasp an object, participants tended to open the relevant effector wider when no visual feedback was provided to adjust for a better grip
*Posture modulates implicit hand maps*	Longo M.R.	2015	Exp	Judged finger posture Over- and under-estimation of landmarks	Body-landmarks localization task (fingers spread apart or squeezed together)	If participants are asked to point to specific locations on their hands with their fingers splayed apart or pressed together, they tend to produce a wider hand representation in the “finger splayed” condition.

### 3.6 Illusion of ownership

One common way to test the ability of the BS to adapt to different situations is to use the Rubber Hand Illusion (RHI) to induce an illusion of ownership. The procedure consists in stroking a prosthetic hand in spatial and temporal synchrony with the participant's concealed hand, until a feeling of ownership over the rubber hand is induced. During the illusion, the BS recalibrates itself so that the participant begins to feel like the rubber hand has been incorporated into their body.

One study demonstrated that the introspective interview is appropriate for the investigation of embodied experience evoked during the RHI. Not only this, but the researchers also predicted that the introspective interview would elicit stronger feelings of ownership over the rubber hand as measured by both self-report and behavioral measures; moreover, this increase in illusory ownership would lead to less variation in self-report responses of subjective experience. Finally, they anticipated that the first-person approach of the introspective interview and interpretative phenomenological analysis would produce a comprehensive and detailed structural account revealing further subcomponents of embodiment. The results showed that, relative to the questionnaire-only group, the introspective-interview group reported significantly stronger subjective feelings of ownership and greater proprioceptive distortion. These findings indicate that introspection during the RHI strengthens the illusion. Additionally, encouraging participants to reflect on their embodied experiences as they occurred also reduced variability in self-report responses. Another study examined the dynamics of the RHI to try and understand the specific conditions under which it stems. Indeed, there has been no inquiry into whether supplementary tactile stimulation on the hand, beyond what is conventionally administered in the Rubber Hand Illusion (RHI), elicits a distinct effect on motor responses. As such, they tried to target the motoric body representation by stimulating the thumb as well as the index finger. As well as showing a RHI-dependent effect on perceptual scaling judgments presumably subserved by the body image, this study for the first time demonstrated a RHI effect on kinematic parameters of a grasping movement. Specifically, they observed effects on both MGA (maximum grip aperture) and PV (peak velocity) during the grasping movement. The subjects opened their hands during the grasping motion in accordance with the aperture of the rubber hand. The effect of the rubber hand's grip aperture was therefore comparable to the effect of the starting grip aperture of the participant's own grasping hand.

In summary, the RHI is an effective way to study body ownership, multisensory integration and perception of self. It has also been used in relation to proprioceptive distortions, showing that greater distortions can emerge from introspection. Also, if the illusion is performed right before a grasping task, the sense of ownership experienced makes it more difficult to open the fingers at the appropriate width, so that participants tend to imitate the same finger position of the rubber hand. One could speculate that grip aperture incongruence reduces peak velocity because the BS primarily relies on visual information to encode limb posture. However, when a conflicting proprioceptive update is introduced, movement speed decreases to adjust the grip aperture to the originally calibrated (visually perceived) size (Table 6).

**Table 6:** Summary of “illusion of ownership” records and their findings.

**Title**	**Authors**	**Year**	**Design**	**Dependent variables**	**Task(s)**	**Key findings**
*Embodied experience: A first-person investigation of the rubber hand illusion*	Lewis E., Lloyd D.M.	2010	Exp	Proprioceptive drift Interpretative phenomenological analysis (IPA)	RHI (Rubber Hand Illusion) Introspective interview	Introspecting during the RHI intensifies the strength of the illusion. Participants showed stronger subjective feelings of ownership and greater proprioceptive distortions.
*How many motoric body representations can we grasp?*	Kammers M.P.M., Kootker J.A., Hogendoorn H., Dijkerman H.C.	2010	Exp	Maximum grip aperture Peak velocity Time to maximum grip aperture Time to peak velocity as a proportion of movement time	RHI (with hands, real and fake, in a grasping position at either the same grip aperture or different) Grasping task (cylinders of different diameters)	Authors found the RHI effect on the kinematic parameters of a grasping movement. Specifically, an effect was observed on both MGA (maximum grip aperture) and PV (peak velocity) during the grasping movement. Participants opened their hand during the grasping motion according to the grip aperture of the rubber hand

### 3.7 Others

The following categories each include one record. For this reason, they have been grouped together and will be discussed in detail nevertheless.

The first one took into consideration the role of sound in shaping our bodily perception. Indeed, action sounds can evoke changes in the internal models of arm morphology involved in facilitating action. Hence, researchers have examined the potential impacts of action sounds on subsequent goal-directed actions. Tajadura-Jiménez et al. (2016) developed an apparatus through which it was possible to induce an illusion of arm elongation. Participants had to tap with their right hand in specific locations on a table, but they received an auditory feedback consistent with a much further tap. This created the illusion of owning a longer limb. The findings of this study indicate that altering the spatial positioning of sounds produced by one's hand influences the kinematics of goal-directed arm movements. Significantly, this discovery presents the initial evidence of auditory-induced recalibration of internal models of body morphology, potentially intended to enhance interactions with the environment. These kinematic changes were characterized by reduced mean and peak amplitudes in the velocity of the reaching movements, and by longer movement times. Remarkably, these changes correspond with the kinematic profile of arm-reaching movements performed by participants with longer arms (Tajadura-Jiménez et al., 2016).

The second study (Thurm et al., 2013) explored the possibility that chronic pain could affect the BS. Starting from the scoping of the literature, the authors pointed out that chronic pain changes the cortical representation of the affected body segment and alters the way the brain perceives the body in space, i.e., changes the BS. Disruption of the cortical representation of the body might be expected to disrupt the BS in the presence of chronic pain. Athletes with chronic pain will present with disturbance of the Body Perception Index (BPI) but normal neuropsychological function. It was anticipated that the athlete group would exhibit noticeable alterations in the BS compared to the control group, but this was not the case. However, women of both groups overestimated their body size dimensions, as has been largely observed in the literature. Authors try to interpret the data by suggesting that it is probably a consequence of the intense sensory input from training practice to the somatosensory cortex, affecting the pain experience and correcting the BS (Thurm et al., 2013; but for a different perspective see Schwoebel et al., 2001).

The last one studied the contribution of vestibular signals in forming and maintaining the BS. According to Paillard (1991), gravitational cues play a role in merging and aligning the diverse reference frames that underlie the BS. Humans have evolved within a consistent gravitational field on Earth, and this physical constraint has influenced the development of our body representations. It has been proposed that vestibular signals generated during body movements are utilized to correct the hand's location and trajectory. Studies suggest that vestibular signals update the BS during manual actions (Bresciani et al., 2002), influencing our execution of tasks and interactions with objects in the surrounding environment. The proposal that vestibular signals play a role in shaping the BS, specifically its metric attributes, finds support in animal electrophysiological research. These investigations reveal that vestibular signals are transmitted to somatosensory areas, including representations of the hand and neck within the primary somatosensory cortex. The results of this study indicate that tactile stimuli applied on the left hand were perceived as longer during Caloric Vestibular Stimulation (CVS) than during a contro-stimulation not activating vestibular receptors and that CVS increased the perceived length and width of the left hand during a task requiring to locate anatomical landmarks on the hand. Collectively, the data suggests that vestibular stimulation had the capacity to alter the immediate representation of body segments, indicating a potential impact of vestibular signals on the neural mechanisms that govern the BS (Lopez et al., 2012).

In summary, these studies looked at the BS with more novel perspectives. In doing so, researchers were able to show that BS plasticity can be induced through the spatial manipulation of sound. After the experimental manipulation of the audio-tactile tapping task, participants performed movements coherent with the perception of having a “longer” arm. This work replicates the seminal study by Cardinali et al. (2009a), who used the training of a tool and had their participants saying that their arm felt longer in preparation for the movement.

Then, authors found a manipulation of body schematics during a vestibular stimulation, which suggests the involvement of vestibular signals on the neural mechanism underlying BS. This means that BS plasticity can be observed following external manipulation of bodily signals, as well as internal.

Finally, through a pilot study it is observed if the BS of athletes with chronic pain would be different than a group of controls (Thurm et al., 2013). The results seem to not confirm this hypothesis, keeping in mind that one important limitation was not having a control group of professional athletes without chronic pain. It seems a condition that is very difficult to obtain since more often than not, professional athletes come up with some kind of chronic pain, consequently it is a topic that would benefit from further investigations (Table 7).

**Table 7:** Summary of “others” records and their findings.

**Title**	**Authors**	**Year**	**Variable tested**	**Design**	**Dependent variables**	**Task(s)**	**Key findings**
*Action sounds modulate arm reaching movements*	Tajadura-Jiménez A., Marquardt T., Swapp D., Kitagawa N., Bianchi-Berthouze N.	2016	Action sounds	Exp	Mean velocity Mean latency Amplitude of the peak velocity and acceleration (of the index finger during the reaching movement)	Reaching task Audio-tactile “tapping” task (adaptation task)	The manipulation of the spatial position of the sounds produced by one's hand has an effect on the kinematics of goal directed arm actions. Moreover, the changes in arm motor behavior observed provide support to the hypothesis of an auditory-driven somatosensory recalibration of the body schema.
*Chronic pain effect on body schema and neuropsychological performance in athletes: A pilot study*	Thurm B.E., Matoso A., Diaz A.C., Paschoalini C., Neves E., Tuunelis R., Kiyomoto H.D., Gama E.F.	2013	Chronic pain	Exp	Body perception index Time required to complete the Grooved Pegboard Test Visual analog scale (for pain)	Image marking procedure (IMP, measures body schema) McGill Pain Questionnaire-Short Form Grooved Pegboard Test (for motor coordination)	There was no difference between the body schema of healthy control and athletes with chronic pain. Women showed an overestimation of body size in both groups.
*Vestibular stimulation modifies the body schema*	Lopez C., Schreyer H.M., Preuss N., Mast F.W.	2012	Vestibular signals	Exp	% stimulus on hand perceived longer than stimulus on forehead Eye movements Mean perceived width and length 233–260	Exp 1: Tactile distance comparison task (two conditions: sham vs. caloric vestibular stimulation CVS) Exp 2: Body-landmarks localization task	Data indicate that vestibular stimulation was able to modify the instantaneous representation of body segments, suggesting an influence of vestibular signals on the neural mechanisms underlying the body schema.

## 4 Discussion

The scoping of the literature revealed many different methods and tasks used to study the plasticity of the arms' BS. The most vastly applied is the use of tools to induce embodiment, so that the tool held feels like a part of one's body. This process makes it possible to reach for further spaces, thus providing better interaction with the environment. It was also possible to observe that there are some differences in the plasticity of the BS through the life cycle. Indeed, before the 4^th^ year of age, children have trouble performing a tactile localization task on their arm, while they are able to perform said task by the 5^th^ and 6^th^ year mark. Also, older adults show greater body distortions, meaning that the representation of hand width decreases while age increases. It was also possible to observe that people with high levels of bodily expertise, had better performances at a landmark localization task overall, and showed better body perception and object integration. Moreover, we also reported that words can become social tools and that they can plastically reshape the reaching space just as ordinary tools do. Moreover, it was shown that visual feedback is necessary to adjust the posture for fine motor skills and that RHI is a valuable instrument to study not only body ownership, but also the BS. Finally, more novel approaches were able to substantiate that sound can be used to give the illusion of having a longer arm, that vestibular stimulations can be implied to successfully manipulate body schematics, and that chronic pain does not seem to influence the proportions of the BS.

Each category outlined in the previous sections highlights situations that facilitate the observation of plasticity in the BS of the arm. Despite observing a wide heterogeneity in the methods and the procedures adopted in the studies object of this review, an attempt at synthesis and comparison is proposed in this paper. It is important though to point out that the vast majority of the papers analyzed in this review refer to the hand or the arm. Only one paper considered the whole body using the Image Marking Procedure (Thurm et al., 2013) and very rarely in literature are the lower limbs mentioned (Laessoe et al., 2017). Future research should focus on other parts of the body to understand if the knowledge we acquired about the upper limbs can be generalized or, alternatively, if the limbs have a unique functioning in respect to the rest of the body. Moreover, it would be useful to shed some light on the differences among the tasks usually adopted in this field of research, pointing out which are the more precise and useful in which circumstance. This will help researchers design their studies and will provide some degree of generalizability of their results.

Although there are evident limits to this work, it still sets an initial base for a revision of the methods implied in these lines of research, to hopefully come to a more structured and integrated set of tools and procedures available to researchers.

Keeping in mind that the methods previously discussed are applicable exclusively to participants with an intact BS, we put forward a theoretical hypothesis for the essential components of a functioning BS, that most certainly will need some experimental validation as a direction for future studies.

A common thread evident in the mentioned studies is that all participants exhibited a competent awareness of proprioception. Indeed, the idea that proprioception should be of great importance for the update of the BS is a long-held hypothesis (Head et al., 1920). Proof from a deafferented patient shows a strong resistance toward tool incorporation. As the patient was unable to integrate the tool and revise the representation of arm length, she acquired a novel grasping strategy. Subsequently, this strategy was employed post-tool use to execute grasping actions with her own hand, resulting in a modified kinematic profile for all associated components of the action (Cardinali et al., 2016). This example poses the limitation of being a single-case study, leaving questions open about the state of the patient's BS before deafferentation and the possible differences that could emerge from confronting different deafferented patients.

Another investigation centered on quantifying the metric aspect of participants' lower limbs to elucidate the influence of vision, touch, and proprioception. The authors underscored that proprioception seemed to contribute significantly to estimations of the overall length of the leg. Specifically, the most precise average estimations of overall length proportions occurred when participants were directed to depend on proprioception for their assessments (i.e., the leg was positioned under a screen), compared to merely imagining its presence or forming judgments regarding a simulated leg (Stone et al., 2018; but for a different perspective see also Longo and Haggard, 2012).

Another common factor that could be hypothesized to be responsible for the ability of the BS to be plastic is the sense of agency. Within cognitive neuroscience, the perception of controlling one's own motor actions and thereby influencing events in the external environment is referred to as the “sense of agency.” By definition, the sense of agency depends on the mental association between an intentional action and its sensory outcome (Haggard, 2017). In one work, researchers tried to manipulate the sense of agency felt by the participants to observe if and how this manipulation would affect BS and PPS. They demonstrated that updates to the BS and PPS took place when the consequences of an action coincided temporally with the participant's movements. However, this occurred in a different spatial position from that anticipated based on the actual hand position. This spatial disparity led to a modulation in both BS and PPS, indicating that these representations are shaped by the accurate and dynamic alignment between intentional bodily movements and their spatial outcomes. It is reasonable to suggest that similar modulations of BS and PPS could occur when individuals manipulate a virtual or physical object that is not hand-shaped or related to the body, based on the observations in the study (D'Angelo et al., 2018). It is worth noting, though, that the link between intention and the action following a manipulation of the timing of a sensory signal is now debated. Gutzeit et al. (2023) suggest in an experimental paper that the effects classically observed are more likely due to procedural confounds than to the sense of agency. In-depth studies on this topic are undoubtedly needed, so the following suggestions should be taken carefully as propositions for future research. Our hypothesis is that BS plasticity can be induced through different strategies, such as the ones investigated and depicted in the previous paragraphs. Following our suggestions, it seems that all of these strategies could be applied effectively only if the participants have a good sense of proprioception and agency. In other words, tool embodiment, words, posture, expertise, sounds, and so on are not causes of plasticity, but merely tools. What we think is more plausible to be granting BS plasticity are proprioception and agency. These considerations inevitably raise a series of questions. Assuming that a robust sense of proprioception and agency is requisite for the expansion of BS boundaries, and considering that the enlargement of BS boundaries also entails an expansion of PPS, does this suggest a concurrent erroneous enlargement of the sense of proprioception and agency? In essence, given that PPS delineates the action space, we raise the query as to whether an enlarged PPS, conceivably influenced by an experimental paradigm, engenders a biased augmentation of the sense of proprioception and agency. If so, this would have serious implications in different fields. The first one that comes to mind is clinical. Indeed, due to its plasticity and its ability to expand and retract in certain given situations, it seems that BS could become a useful tool for implementing clinical protocols. Think not only amputees but also eating disorder patients. Bodily dysmorphism could be gradually addressed by making the patient more comfortable with the idea of having longer, fuller limbs. It would be also beneficial to work on reclaiming a good sense of agency over one's body, making patients aware that they have the power to act and make decisions for themselves, instead of succumbing to the idea that changing something about their lives would lead to tragic consequences. This notion would also be applicable to individuals afflicted with mood and anxiety disorders. Another rationale for investigating this phenomenon pertains to its potential implications for studies concerning decision-making processes. Specifically, it prompts inquiry into whether beliefs regarding personal agency (i.e., the conviction of one's capacity to influence the environment) are strengthened by an expanded PPS, which delineates the sphere of action. Consequently, a pertinent inquiry arises regarding the potential alterations in decision-making strategies. Might an extension of PPS lead to an overestimation of personal capabilities? In other words, could this engender a propensity toward riskier decision-making in situations characterized by uncertainty? On a broader scope, what interrelation exists between the surrounding environment and an individual's aptitude for decision-making? We think it would be valuable to better investigate these mechanisms to provide deeper knowledge about certain (still debated) aspects of decision making, such as moral dilemmas, gambling, economics and much more.

## Data Availability

The original contributions presented in the study are included in the article/supplementary material, further inquiries can be directed to the corresponding author.
